# Evaluating the efficacy and safety of various flaps in Autologous Breast Reconstruction: a Bayesian network meta-analysis

**DOI:** 10.3389/fmed.2024.1440139

**Published:** 2024-09-20

**Authors:** Ling Li, Di Wu, Xiaohai Zhu

**Affiliations:** ^1^Department of Plastic Surgery, Changzheng Hospital, Second Military Medical University, Shanghai, China; ^2^Department of Cardiothoracic Surgery, Changzheng Hospital, Second Military Medical University, Shanghai, China

**Keywords:** Autologous Breast Reconstruction, flap, network meta-analysis, efficacy, safety

## Abstract

**Aim:**

This analysis evaluates the efficacy and safety of different flap techniques for Autologous Breast Reconstruction by collecting all clinical trials employing these methods and applying a Bayesian network meta-analysis.

**Materials and methods:**

We systematically searched PubMed, Embase, and Web of Science for relevant literature, focusing on outcomes such as total flap loss, donor site wound dehiscence, secondary corrections at the donor site, psychosocial well-being, satisfaction with breasts, and sexual well-being.

**Results:**

Our analysis included 10 clinical studies involving 871 patients across six flap techniques. In terms of total flap loss, the TUG flap showed the highest SUCRA value (89.6%), followed by the TMG flap (64.8%). For donor site wound dehiscence, the DIEP flap ranked highest with a SUCRA value of 60.1%, followed by the PAP flap (48.6%). In secondary corrections at the donor site, the PAP flap was the leader (95.5%), followed by the DIEP flap (52.5%) and the TMG flap (41.4%). For outcomes related to psychosocial well-being, satisfaction with breasts, and sexual well-being, the Four-flap technique consistently ranked highest (81.3, 85.0, and 88.4%, respectively).

**Conclusion:**

Various flap techniques in Autologous Breast Reconstruction each present distinct benefits and risks. The Four-flap technique shows significant advantages in patient satisfaction, while the TUG flap excels in reducing total flap loss.

## 1 Introduction

Breast cancer is a common malignancy and the leading cause of cancer-related death among women worldwide (1). In 2023, breast cancer accounted for 12.4% of all cancer cases and 23.8% of all female cancers, with over 2.3 million new diagnoses and approximately 660,000 deaths, representing 6.9% of all cancer deaths and 15.4% of female cancer fatalities (1). According to the American Cancer Society, an estimated 310,720 new cases of invasive breast cancer in women will be diagnosed in the United States in 2024, and the rise in incidence rates have increased by 0.6% per year (2, 3).

Surgical treatment remains the primary modality for managing breast cancer, with mastectomy being the most common surgical procedure utilized clinically (3). Mastectomy significantly impacts the psychological health and social functioning of female patients, as changes in physical appearance after surgery often lead to various negative emotions (4). Research by Skrzypulec et al. (5) showed significant increases in depression and anxiety levels post-mastectomy. Breast reconstruction plays a crucial role in breast cancer treatment, aiding patients in regaining their physical image and self-esteem, thereby enhancing their quality of life.

Breast reconstruction is extremely important in the treatment of breast cancer, as it helps patients regain their physical image and self-esteem, thereby improving their quality of life (6). Autologous Breast Reconstruction, which uses the patient’s own tissues to create a new breast, is widely adopted. This method minimizes the risks associated with implants and provides a more natural appearance and feel. Current popular techniques for breast reconstruction include the Deep Inferior Epigastric Perforator (DIEP) flap, Profunda Artery Perforator (PAP) flap, muscle-sparing Transverse Rectus Abdominis Myocutaneous (TRAM) flap, Inferior Gluteal Artery Perforator (IGAP) flap, Transverse Myocutaneous Gracilis (TMG) flap, and the free Transverse Upper Gracilis (TUG) flap (7–10).

The DIEP flap is a reliable breast reconstruction technique commonly chosen by patients due to fewer complications at the donor site (11). However, absolute contraindications include prior abdominal surgery or liposuction, and smoking within 1 month before surgery (12). The PAP flap, considered an alternative to the DIEP flap, is better suited for patients with severe abdominal scarring, insufficient soft tissue volume, those who prefer to avoid a prominent abdominal scar, or those planning for pregnancy soon (10, 13). However, it is generally considered that donor site complications in the PAP flap are higher than in the DIEP flap.

Although these breast reconstruction techniques improve patient quality of life postoperatively, high-quality randomized controlled trials assessing postoperative outcomes like flap loss rate, donor site wound healing, psychological health, and patient satisfaction with surgical results are lacking (14). This study aims to evaluate the outcomes of all breast reconstruction techniques following mastectomy, indirectly comparing the efficacy and safety of treatment options based on a Bayesian framework, and determining the optimal breast reconstruction strategy through systematic review and meta-analysis.

## 2 Materials and methods

### 2.1 Literature search

A systematic review of literature was performed on May 05, 2024, following PRISMA guideline (15). English databases, including PubMed, Embase, and Web of science, were searched for articles. Our search strategy used the following Mesh terms and keywords: “mammaplasty,” “breast reconstruction,” “profunda artery perforator flap,” “PAP flap,” and “PAPF,” linked with Booleans operators (Table 1). Additionally, we performed manual searches by examining all references, exploring gray literature, and reviewing theses, government documents, letters, abstracts, minutes of meetings, and research reports to mitigate potential publication bias.

**Table 1:** Search strategy.

Database	Date	Search query	No. of articles
PubMed	May 05, 2024	((mammaplasty[MeSH Terms]) OR (((Mammaplast*[Title/Abstract]) OR (Mammoplast*[Title/Abstract])) OR (breast reconstruction*[Title/Abstract]))) AND ((profunda artery perforator flap*[Title/Abstract]) OR (PAP flap*[Title/Abstract]))	99
Embase	May 05, 2024	((‘breast reconstruction’/exp) OR (mammaplast*:ab,ti OR mammoplast*:ab,ti OR ‘breast reconstruction*’:ab,ti)) AND (‘profunda artery perforator flap’:ab,ti OR ‘profunda artery perforator flap*’:ab,ti OR ‘pap flap*’:ab,ti)	78
Web of science	May 05, 2024	((TS = (mammaplasty)) OR (((TS = (mammaplast*)) OR TS = (Mammoplast*)) OR TS = (breast reconstruction*))) AND ((TS = (profunda artery perforator flap*)) OR TS = (PAP flap*))	112

### 2.2 Selection criteria

Eligibility studies in our meta-analysis satisfied the following criteria: (1) Study design: clinical trials, including randomized controlled trial, cohort study, case–control study, or comparative study; (2) Patients: adults undergoing breast reconstruction; (3) Interventions and controls: different flaps; (4) Outcomes: measuring at least one of the following outcomes: total flap loss, donor site wound dehiscence, secondary corrections at the donor site, psychosocial well-being, satisfaction with breasts, and sexual well-being. Exclusion Criteria: (1) Trials based on the same group of patients at different times; (2) Trials that do not include the required outcome measures; (3) Reviews, case reports, or animal studies.

All included studies were double-checked by two reviewers to ensure that the data from the included trials were up-to-date.

### 2.3 Data extraction and quality assessment

Relevant articles retrieved from the database search were independently scrutinized by title and abstract by two investigators. Subsequently, data were independently collected using a standardized Excel data extraction sheet. In cases of disagreement, the article was reviewed by a third investigator, and a resolution was reached following a discussion among the three authors.

Demographic variables, primary outcomes, and secondary outcomes were recorded. Demographic variables included the authors, publication date, country of origin, mean body mass index (BMI), average flap weight, and pedicle length. Additionally, the required outcome measures were extracted.

For non-randomized trials, we employed the modified Newcastle-Ottawa Scale (NOS) to assess the methodological quality (16). This method comprised of three items to evaluate the quality of a non-RCT trial. The total score of this method was 9 points, and higher points indicated high quality. Studies with a score of more than 5 points were regarded as high quality.

### 2.4 Statistical analysis

Bayesian network meta-analysis was conducted using STATA 17. For dichotomous outcome measures, odds ratios (OR) and their 95% confidence intervals (95% CI) were calculated to estimate the pooled effect sizes. For continuous outcomes with non-uniform measurement units, the standardized mean difference (SMD) and its 95% CI were used to compute the pooled effect sizes, thereby eliminating bias caused by unit differences. Network graphs were drawn, and if a closed loop structure was present within the network, loop inconsistency tests were conducted to assess the consistency of the outcomes. If the *p*-value was greater than 0.05, indicating good consistency between direct and indirect evidence, a consistency model was used. Otherwise, sources of heterogeneity were explored through subgroup analysis and regression analysis. For open loop structures within the network graph, a consistency model was chosen. Subsequently, cumulative ranking curves (SUCRA) (17) were plotted to determine the best flap techniques in Autologous Breast Reconstruction. Lastly, comparison-adjusted funnel plots were utilized to detect publication bias and small-study effects.

## 3 Results

### 3.1 Literature search

**Figure 1:**
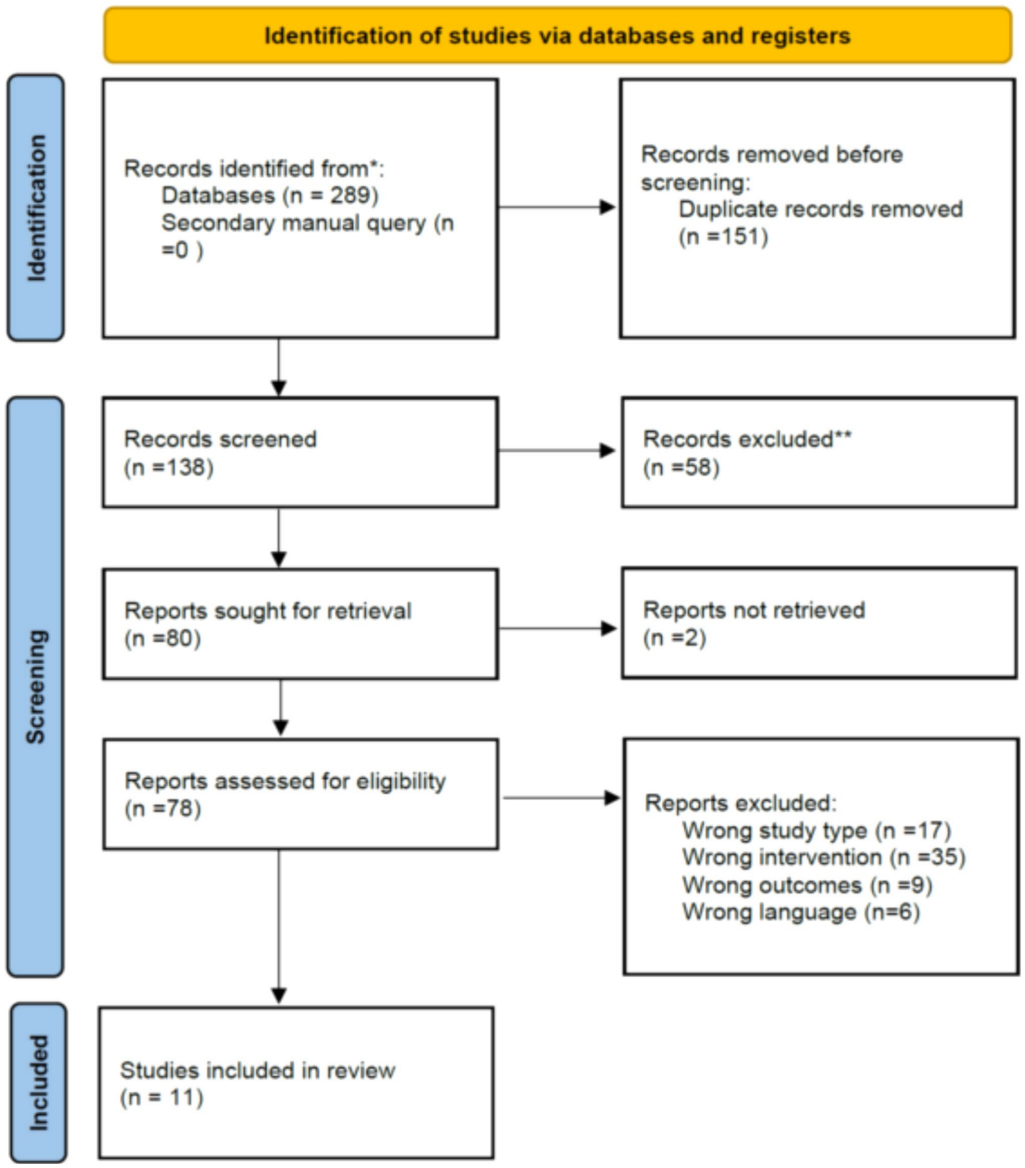
Literature search and screening flowchart.

A total of 289 studies were identified through database searches. After removing 151 duplicate records, 138 studies remained for title and abstract review. Of these, 58 were excluded for various reasons. Subsequently, the remaining 78 studies were fully read for eligibility assessment, and then a total of 11 studies that fully met the inclusion criteria (10, 18–27) (Figure 1).

### 3.2 Clinical characteristics of included studies

Table 2 provides summaries of the key features of the studies included in the meta-analysis. Across these 11 studies, a total of 871 patients were enrolled, each undergoing one of six different flap techniques: PAP flap, DIEP flap, TMG flap, IGAP flap, Four-flap, and TUG flap. Two of the studies were conducted in the United States, United Kingdom, Austria, and Korea, respectively, while two additional studies were conducted in Germany and China, respectively. The mean age of patients using PAP flaps ranged from 39.1 to 51.4 years, compared to 41.3 to 51.5 years for those using other flaps. The mean BMI for patients using PAP flaps ranged from 20.6 to 25 kg/m^2^, whereas for those using other flaps, it ranged from 19.6 to 40 kg/m^2^.

**Table 2:** Clinical characteristics of included studies.

Author	Publication Year	Country	Intervention	No. of Patients	Age (Mean ± SD)	BMI (Mean ± SD)	Flap weight	Control	No. of Patients	Age (Mean ± SD)	BMI (Mean ± SD)	Flap weight	NOS score
Augustin et al. (18)	2023	Austria	27PAP Flaps	9	43.6 ± 7.4	21.6 ± 2.3	327.7 ± 108.2 cc	28DIEP Flaps	10	41.3 ± 6.7	25.3 ± 3.7	565.2 ± 207.4 cc	5
Augustin et al. (19)	2022	Austria	28PAP Flaps	18	43.6 ± 7.4	21.6 ± 2.3	327.7 ± 108.2 cc	25TMG Flaps	22	NA	NA	NA	5
Chan et al. (20)	2023	China	17PAP Flaps	17	39.6 ± 8.4	20.6 ± 1.9	229.5 ± 42.3 g	11DIEP Flaps	11	45.3 ± 6.6	19.6 ± 1.3	251.7 ± 32.2 g	6
Haddock et al. (21)	2022	US	62PAP Flaps	31	47.4 ± 10	24.7 ± 4.1	NA	312DIEP Flaps	56	51.48 ± 9.2	40 ± 5.6	NA	6
			62PAP Flaps	31	47.4 ± 10	24.7 ± 4.1	NA	124Four Flaps	56	50.4 ± 9.3	25.8 ± 3.7	NA	
Hunter et al. (10)	2015	UK	22PAP Flaps	13	48(32–61)	21.6(19.0–31)	242.0(132–455) g	54TUG Flaps	39	48(35–61)	22.3(19.4–27)	294.9(149–500) g	5
Jo et al. (22)	2022	Korea	43PAP Flaps	28	39.9 ± 1.5	22.2 ± 0.5	308.9 ± 19.8 g	192 DIEP Flaps	192	47.9 ± 0.6	24.1 ± 0.3	410.9 ± 11.3 g	6
Kim et al. (23)	2023	Korea	43PAP Flaps	27	39.1 ± 7.3	22.7 ± 2.8	229.1 ± 86.5 g	99DIEP Flaps	95	47.4 ± 7.7	24.3 ± 3.4	415.5 ± 153.3 g	6
Lee et al. (24)	2022	US	41PAP Flaps	30	51.4 ± 9.4	NA	NA	91DIEP Flaps	60	51.4 ± 9.3	NA	NA	6
Murphy et al. (25)	2022	UK	73PAP Flaps	51	45 ± 8.7	25 ± 2.7	257(231, 287)	47IGAP Flaps	43	47 ± 7.1	22 ± 2.2	335(305, 370)	6
Teotia et al. (26)	2020	USA	59 PAP Flaps	59	49.07	37.48	NA	82DIEP Flaps	82	54.79	27.23	NA	6
Varnava et al. (27)	2023	German	85PAP Flaps	73	45.9 ± 12.4	23.1 ± 3.6	NA	122 DIEP Flaps	87	51.1 ± 10.3	28.0 ± 4.7	NA	7

PAP, profunda artery perforator; BMI, body mass index; SD, standard deviation; NA, not available; DIEP, deep inferior epigastric perforator; IGAP, inferior gluteal artery perforator; TMG, transverse myocutaneous gracilis; TUG, free transverse upper gracilis.

### 3.3 Literature quality assessment

The methodological assessment for cohort studies showed that, the NOS score in each study was greater than 5 points, indicating that they were of high quality (Table 2).

### 3.4 Network evidence graph

**Figure 2:**
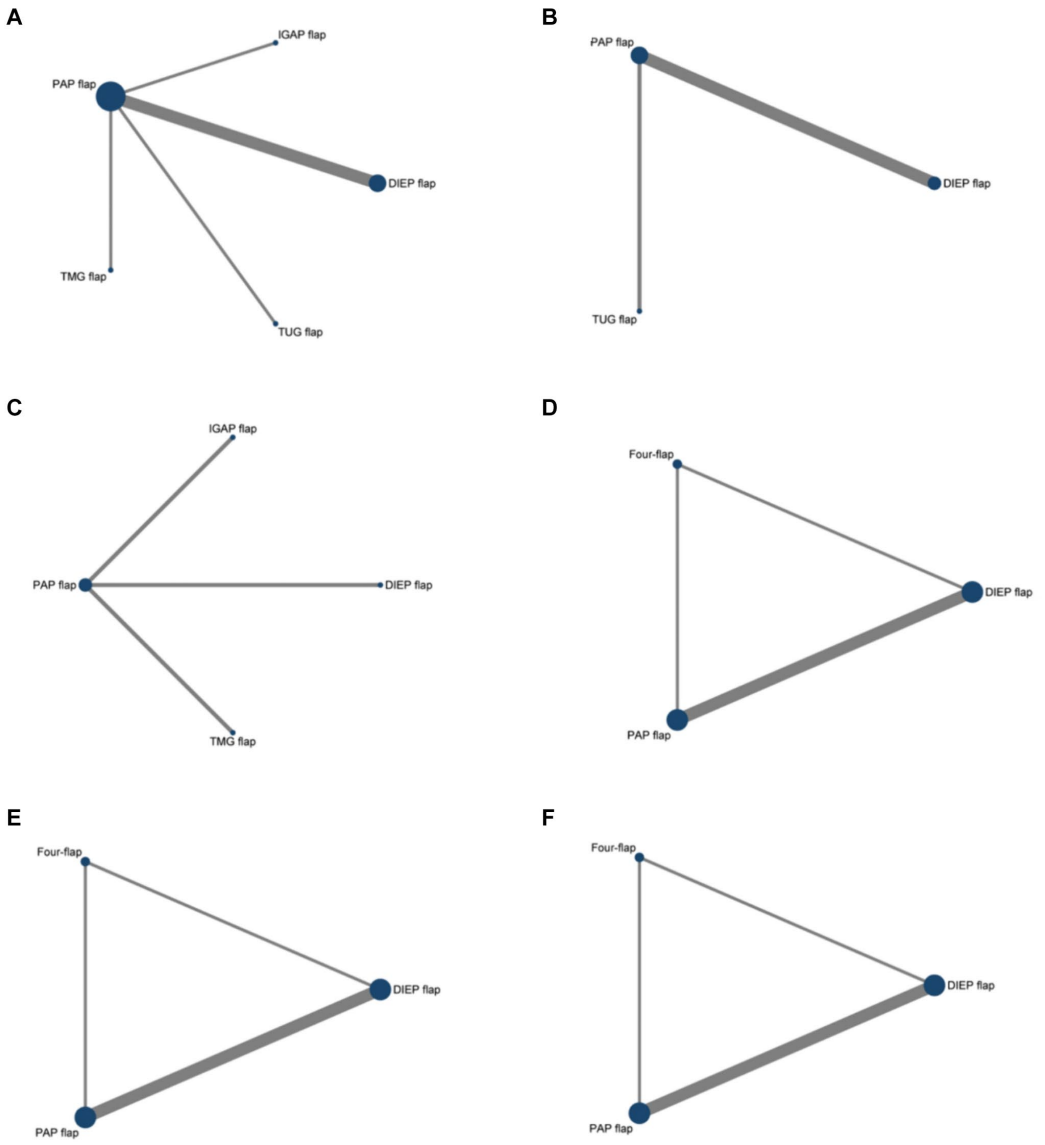
Network graph comparing the efficacy and safety of various flaps in Autologous Breast Reconstruction. **(A)** Total flap loss, **(B)** donor site wound dehiscence, **(C)** secondary corrections donor site, **(D)** psychosocial well-being, **(E)** satisfaction with breasts, **(F)** sexual well-being.

The network evidence graph presented by the network meta-analysis consists of nodes, each representing a different intervention. The size of a node corresponds to the sample size of the intervention, with larger nodes indicating larger sample sizes. Lines between the nodes represent direct comparisons between interventions, with the thickness of the lines reflecting the number of direct comparison studies. Thicker lines indicate more evidence from direct comparisons, whereas thinner lines suggest fewer studies. The absence of a line between two nodes indicates that there are no direct comparisons between those interventions, necessitating the use of network meta-analysis to derive indirect evidence. A network comparison diagram showing the relationships between studies for six different outcome measures can be seen in Figure 2.

### 3.5 Efficacy

Since the network evidence graphs for psychosocial well-being, satisfaction with breasts, and sexual well-being are all open loop, a consistency model was applied for the network meta-analysis.

#### 3.5.1 Psychosocial well-being

**Figure 3:**
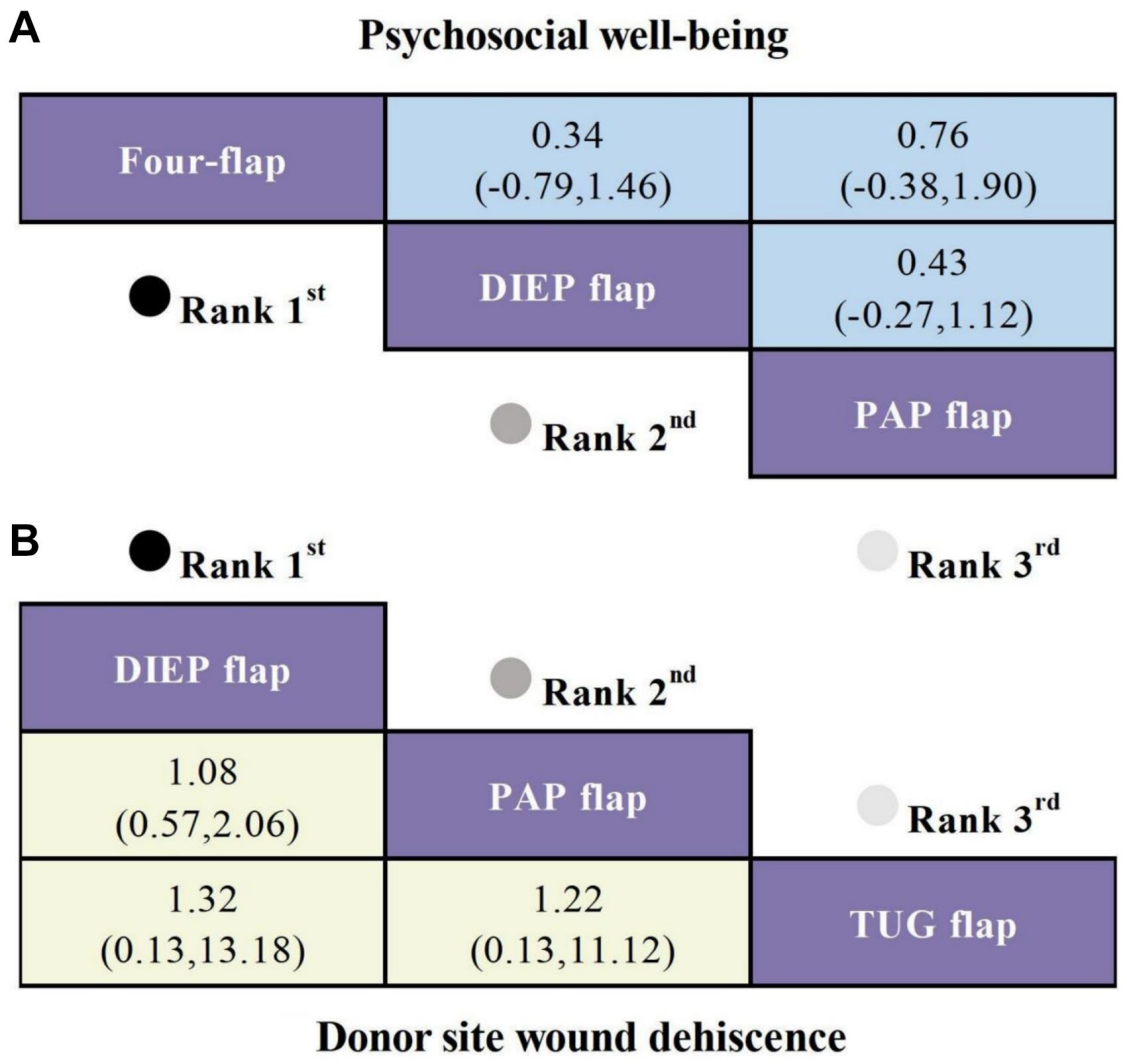
SUCRA curves based on Bayesian network meta-analysis comparing the efficacy and safety of different flaps in Autologous Breast Reconstruction. **(A)** SMD and 95% CI for psychosocial well-being (depicted in the blue upper triangular area). **(B)** OR and 95% CI for donor site wound dehiscence (depicted in the yellow lower triangular area).

Four studies included psychosocial well-being as an outcome, involving three types of flaps. Compared to the PAP flap, the Four-flap showed the least reduction in psychosocial well-being scores (SMD = 0.76, 95% CI: −0.38, 1.90), though the difference was not statistically significant (Figure 3A).

#### 3.5.2 Sexual well-being

**Figure 4:**
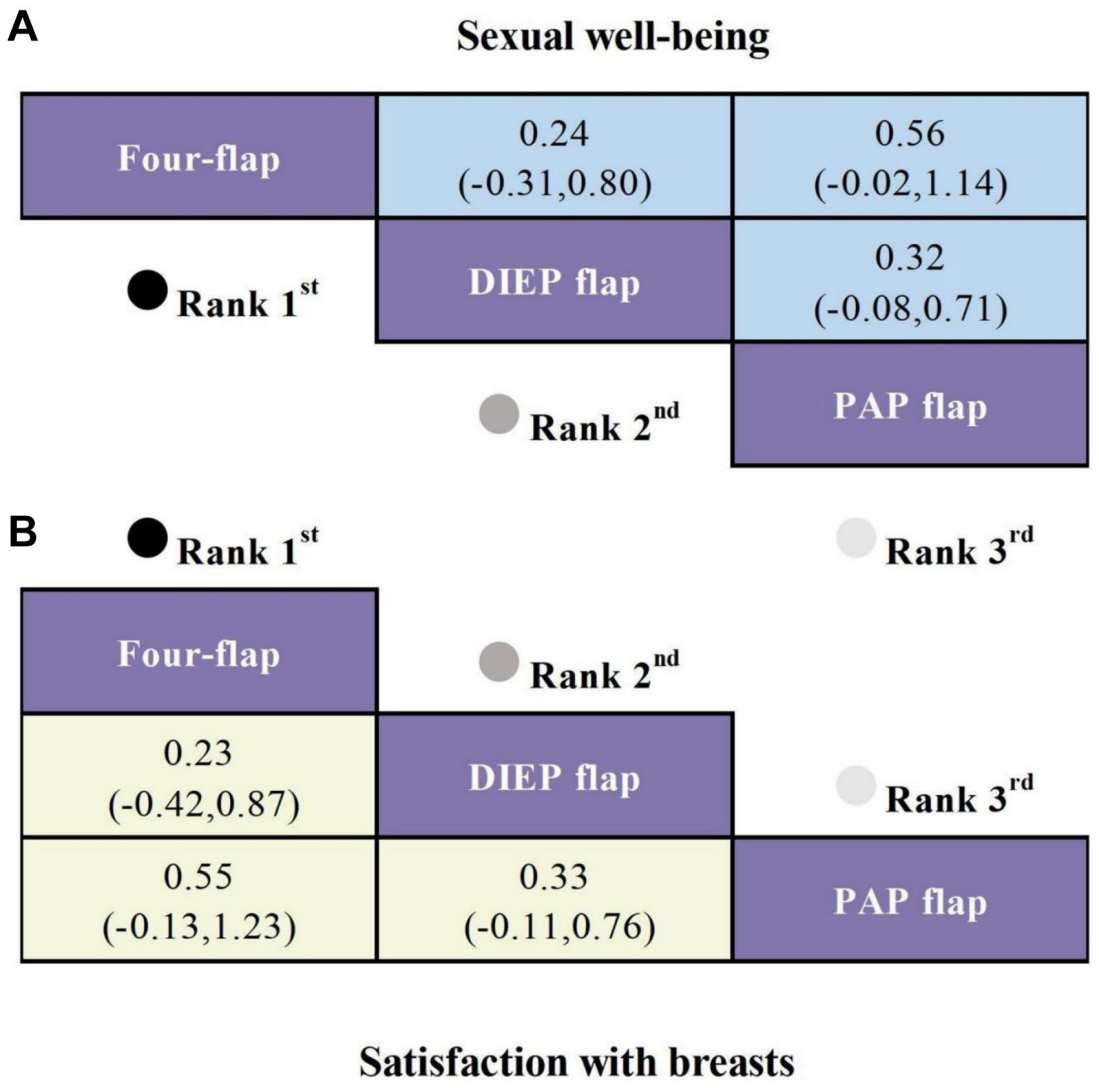
SUCRA curves from Bayesian network meta-analysis comparing the efficacy of different flaps in Autologous Breast Reconstruction. **(A)** SMD and 95% CI for sexual well-being (shown in the blue upper triangular area). **(B)** SMD and 95% CI for satisfaction with breasts (shown in the yellow lower triangular area).

Four studies reported on sexual well-being as an outcome, involving three different types of flaps. Compared to the PAP flap, the Four-flap showed the smallest reduction in sexual well-being scores (SMD = 0.56, 95% CI: −0.02, 1.14), followed by the DIEP flap (SMD = 0.32, 95% CI: −0.08, 0.71), although these differences were not statistically significant (Figure 4A).

#### 3.5.3 Satisfaction with breasts

Four studies reported on satisfaction with breasts as an outcome, involving three different types of flaps. Compared to the PAP flap, the Four-flap demonstrated the smallest decrease in satisfaction (SMD = 0.55, 95% CI: −0.13, 1.23), followed by the DIEP flap (SMD = 0.33, 95% CI: −0.11, 0.76), although these differences were not statistically significant (Figure 4B).

### 3.6 Safety

The network graphs for psychosocial well-being, satisfaction with breasts, and sexual well-being are all closed loops. An inconsistency model was employed for testing, with results showing *p* > 0.05, thus confirming consistency in the studies. A consistency model was used for the network meta-analysis.

#### 3.6.1 Total flap loss

**Figure 5:**
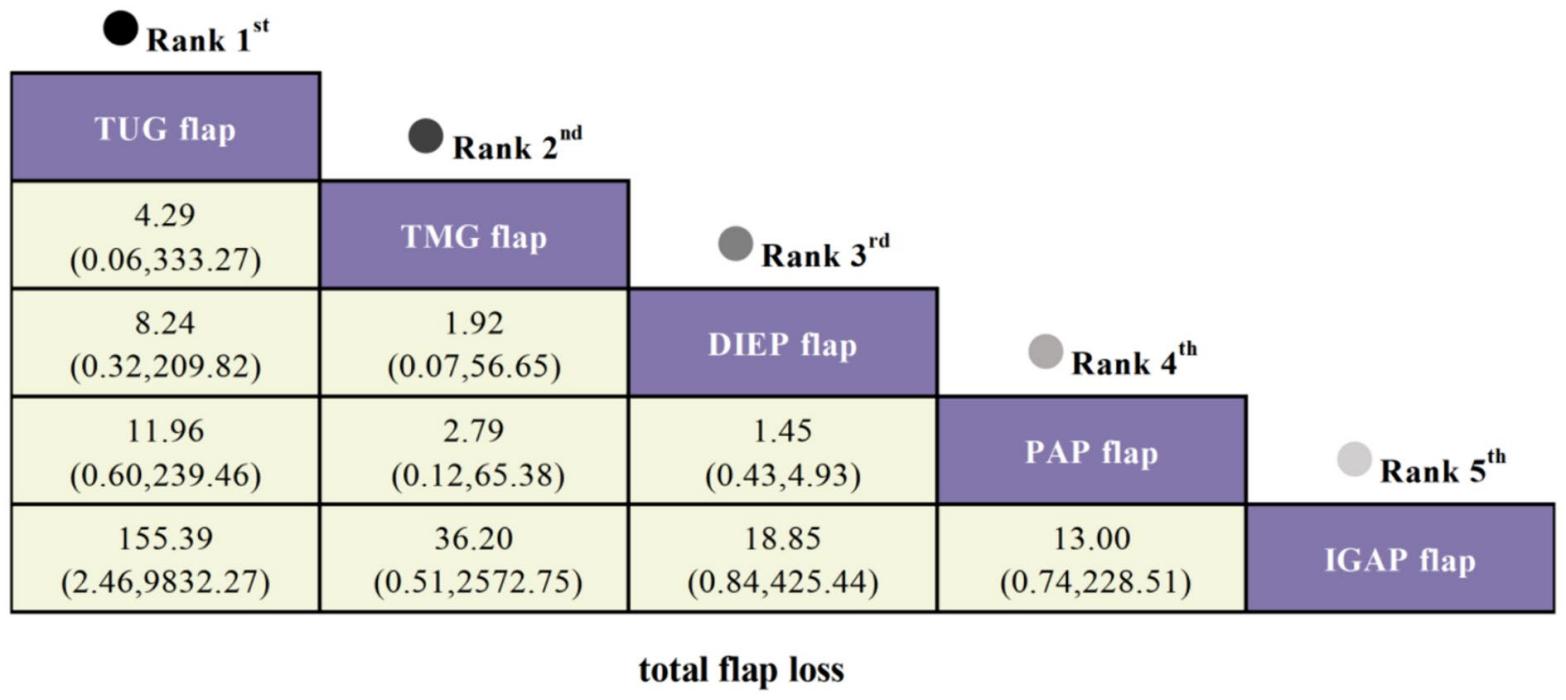
SUCRA curves from Bayesian network meta-analysis comparing total flap loss among different flaps in Autologous Breast Reconstruction.

Seven studies included total flap loss as an outcome, covering five types of flaps. Compared to the IGAP flap, the TUG flap exhibited the lowest risk of total flap loss (OR = 155.39, 95% CI: 2.46, 9832.27), which was statistically significant. This was followed by the TMG flap (OR = 36.2, 95% CI: 0.51, 2572.75) and DIEP flap (OR = 18.85, 95% CI: 0.84, 425.44), though these findings did not reach statistical significance (Figure 5).

#### 3.6.2 Donor site wound dehiscence

Four studies reported donor site wound dehiscence as an outcome, involving three different types of flaps. Compared to the TUG flap, the DIEP flap exhibited the lowest risk of donor site wound dehiscence (OR = 1.32, 95% CI: 0.57, 2.06), followed by another flap (OR = 1.22, 95% CI: 0.13, 11.12), although these differences were not statistically significant (Figure 3B).

#### 3.6.3 Secondary corrections at donor site

**Figure 6:**
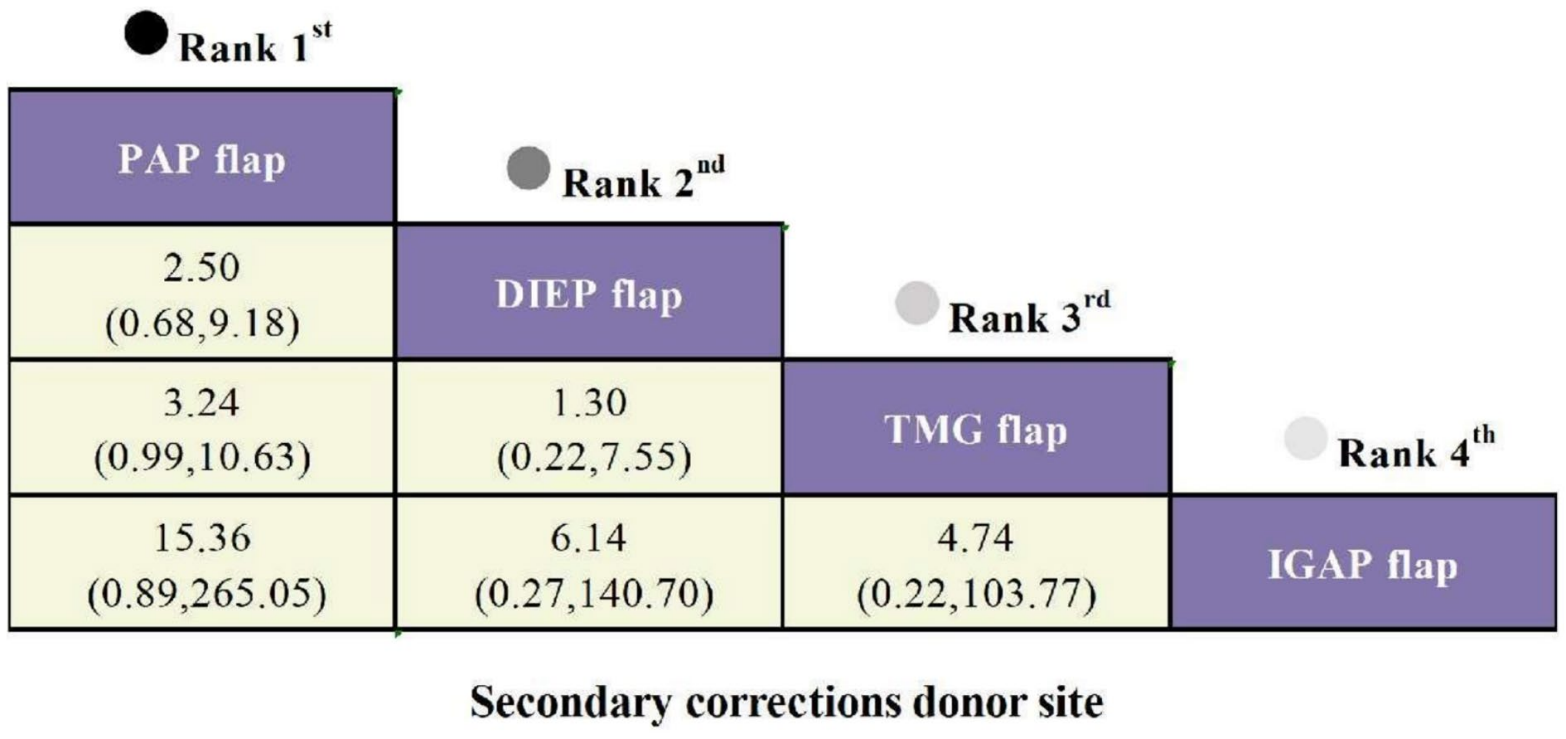
SUCRA curves from Bayesian network meta-analysis comparing secondary corrections at donor site among different flaps in Autologous Breast Reconstruction.

Three studies reported secondary corrections at the donor site as an outcome, involving four types of flaps. Compared to the IGAP flap, the PAP flap had the lowest risk of secondary corrections (OR = 15.36, 95% CI: 0.89, 265.05), followed by the DIEP flap (OR = 6.14, 95% CI: 0.27, 140.70) and the TMG flap (OR = 4.74, 95% CI: 0.22, 103.77), though none showed statistically significant differences (Figure 6).

### 3.7 Rankings

Rankings were determined by comparing the Surface Under the Cumulative Ranking Curve (SUCRA), where the SUCRA values range from 0 to 100% (Table 3). A higher SUCRA value indicates better performance of the flap.

**Table 3:** Cumulative probability rankings.

	Total Flap Loss	Donor site wound dehiscence	Secondary corrections donor site	Psychosocial well-being	Satisfaction with breasts	Sexual well-being
TUG flap	0.896	0.413	–	–	–	–
TMG flap	0.648	–	0.414	–	–	–
DIEP flap	0.534	0.601	0.525	0.581	0.583	0.569
PAP flap	0.388	0.486	0.955	0.106	0.067	0.047
IGAP flap	0.034	–	0.105	–	–	–
Four-flap	–	–	–	0.813	0.85	0.884

PAP, profunda artery perforator; DIEP, deep inferior epigastric perforator; IGAP, inferior gluteal artery perforator; TMG, transverse myocutaneous gracilis; TUG, free transverse upper gracilis.

Results show that in the outcome measure of Total Flap Loss, the TUG flap had the highest SUCRA value at 89.6%, followed by the TMG flap at 64.8% (Table 3). For the outcome measure of Donor Site Wound Dehiscence, the DIEP flap had the highest SUCRA value at 60.1%, followed by the PAP flap at 48.6%. In the outcome measure of Secondary Corrections at Donor Site, the PAP flap ranked first with a SUCRA value of 95.5%, followed by the DIEP flap at 52.5% and the TMG flap at 41.4%. For the outcome measures of Psychosocial Well-being, Satisfaction with Breasts, and Sexual Well-being, the Four-flap consistently ranked first (81.3, 85.0, and 88.4%).

### 3.8 Publication bias

**Figure 7:**
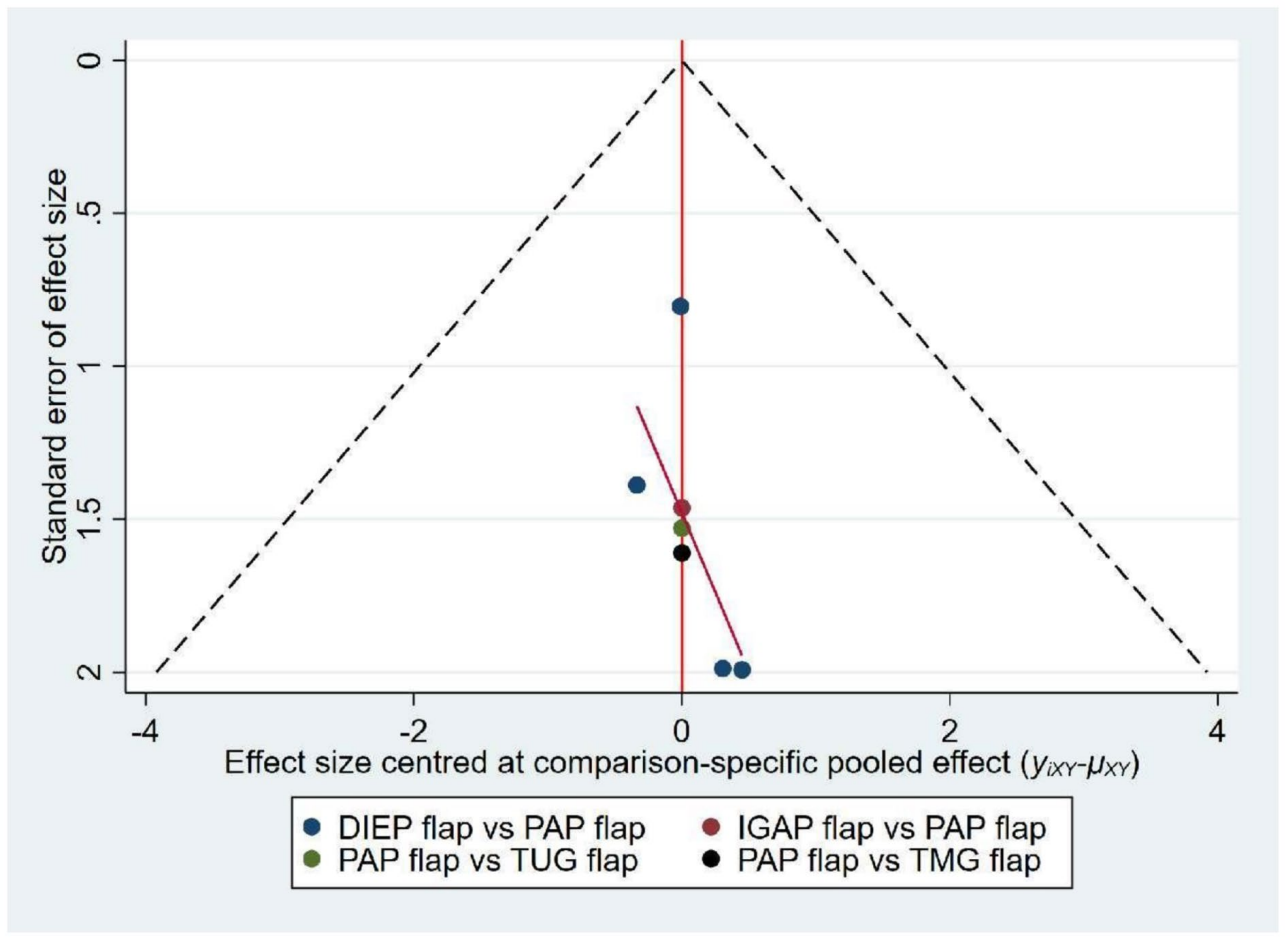
Funnel plot.

The funnel plot, as illustrated, shows that the data points are symmetrically and uniformly distributed within the funnel plot. The overall results indicate no significant publication bias, suggesting that the findings are robust and reliable (Figure 7).

## 4 Discussion

To our knowledge, this Bayesian network meta-analysis is the first to simultaneously evaluate the efficacy and safety of multiple types of flaps, including PAP, DIEP, TMG, TUG, IGAP, and Four-flap. Each flap type has its unique benefits and risks. Our study conducted a quantitative synthesis, revealing the efficacy and safety of different flap types, including the following: (1) The TUG flap exhibited the lowest risk of total flap loss, indicating higher reliability in certain patient populations. (2) Patients with the Four-flap reported the highest satisfaction levels. (3) The PAP flap excelled in secondary corrections at the donor site. (4) Patients with PAP flaps scored lower in psychological well-being, breast satisfaction, and sexual satisfaction compared to other groups.

The four-flap technique typically utilizes the patient’s own adipose and skin tissue for breast reconstruction, resulting in a breast that closely mimics the natural appearance and feel (28). This method allows for precise adjustments to the breast’s shape and size, enhancing symmetry with the contralateral breast and thereby improving aesthetic harmony (29). By avoiding the use of foreign implants, such as prostheses, the four-flap technique reduces the risk of complications associated with implants, including rejection reactions and implant rupture (30). Although the four-flap surgery May involve a more complex procedure and longer recovery time, the reduced use of foreign materials May contribute to a more positive postoperative self-perception in patients (31). This explains why patient satisfaction is highest with four-flap procedures.

Breast reconstruction patients often experience significant psychological stress. The use of autologous tissue, particularly when it achieves an outcome that closely resembles a natural breast, can provide substantial psychological comfort and boost the patient’s confidence, which supports the conclusions of our study. Compared to existing meta-analyses, which often focus on fewer flap types or non-autologous methods—sometimes overlooking the PAP flap—our study identifies its particular benefits for patients with prior abdominal surgeries (8). In terms of psychosocial well-being, satisfaction with breasts, and sexual well-being, patients with the PAP flap scored lower than other groups, possibly because those choosing the PAP flap tend to be slimmer, and the tissue volume provided by the PAP flap is somewhat limited (32). Breast reconstruction is more than just physical restoration; it profoundly affects a woman’s self-esteem, body image, and overall quality of life. Our findings indicate that selecting the appropriate breast reconstruction flap technique can significantly enhance life satisfaction after mastectomy.

The TUG flap Autologous Breast Reconstruction primarily utilizes the gracilis muscle from the inner thigh along with its associated skin and adipose tissue, making it suitable for patients lacking sufficient abdominal tissue (17). The gracilis muscle used in the TUG flap is supplied by branches of the deep artery, which reduces the risk of postoperative flap ischemia and consequently lowers the flap loss rate (33). The skin and fat from the inner thigh have a texture similar to that of breast tissue, making the TUG flap aesthetically more compatible with the natural breast. Additionally, the good elasticity and softness of the inner thigh tissue enhance the adaptability of the transplanted flap, promoting its survival (34). Compared to other methods requiring substantial tissue transfer, such as the DIEP flap, the TUG flap requires less tissue, thereby reducing surgical invasiveness and associated complication risks, ultimately decreasing the overall flap loss rate (35).

Despite the comprehensiveness of our analysis and discussion, there are limitations to this study. Firstly, although an extensive search of English-language databases was conducted, clinical trials focusing on patient satisfaction outcomes were limited to only three types of flaps. Secondly, seven of the 11 included studies were conducted in Europe, reducing the general applicability of the results, which May not be as relevant to populations in Asia and Africa. Thirdly, due to the limited number of studies and the minor differences between various flap techniques, some outcomes showed no statistically significant differences. Future research should include more uniform and rigorous study designs and long-term follow-up data to further validate our findings. Fourthly, some outcomes were analyzed using data from only one or two studies, which May introduce bias due to the small sample size. Consequently, these results should be interpreted with caution. Additional studies are necessary to validate our findings and ensure their reliability. Lastly, not all included studies reported flap volume or degrees of patient satisfaction. The absence of these important variables limits our ability to fully assess the outcomes of different flap techniques. Future studies should aim to include detailed reporting on flap volume and comprehensive measures of patient satisfaction to provide a more complete understanding of the effectiveness of these techniques.

While our Bayesian network meta-analysis highlights the relative strengths and weaknesses of each flap technique, we acknowledge that the results do not definitively establish the superiority of any single technique. This is due to the inherent variability in patient characteristics and clinical circumstances that influence the choice of flap. However, we have provided practical guidelines based on the findings to assist clinicians in selecting the most appropriate flap technique for individual patients.

In conclusion, various flap techniques in Autologous Breast Reconstruction each present distinct benefits and risks. The Four-flap technique shows significant advantages in patient satisfaction, while the TUG flap excels in reducing total flap loss.

## Data Availability

The raw data supporting the conclusions of this article will be made available by the authors, without undue reservation.
